# Daily life after healing of a venous leg ulcer: A lifeworld phenomenological study

**DOI:** 10.1080/17482631.2022.2054080

**Published:** 2022-03-20

**Authors:** Marcus Rosenburg, Cecilia Fagerström, Hanna Tuvesson, Gunilla Lindqvist

**Affiliations:** a Faculty of Health and Life Sciences, Department of Health and Caring Sciences, Linnaeus University, Växjö, Sweden; b School of Health and Welfare, Department of Health and Nursing, Halmstad University, Halmstad, Sweden; c Department of Research, Region Kalmar County, Sweden; d Faculty of Health and Life Sciences, Departement of Health and Caring Sciences, Linnaeus University, Kalmar, Sweden

**Keywords:** Life change events, life experiences, qualitative research, varicose ulcer, wound healing

## Abstract

**Purpose:**

Venous leg ulcer is a recognized condition, affecting people globally. Ulcers mainly affect the elderly and recurrences are not uncommon. There is knowledge about life with venous leg ulcers, but the situation after healing is unexplored. This paper explores and describes meanings of experiences of daily life after healing of a hard-to-heal venous leg ulcer.

**Methods:**

Lived experiences of 15 individuals with healed hard-to-heal venous leg ulcers generated data for this study. Interviews were recorded for analysis using a reflective lifeworld research approach. An essence emerged, further described by its constituents.

**Results:**

Memories of a difficult time with leg ulcer were ever present, in a way becoming part of the self. A striving for control in daily life entailed a struggle to do what was best for the own body. After healing, a new normal emerged in daily life, a reality that encompassed the risk for a new ulcer. The body had changed physically, with marks alongside those from ageing, in a life that still went on.

**Conclusions:**

For those who had healed from a venous leg ulcer, life had changed. Even if they referred to life as normal, it was not the same normal as before.

## 2. Introduction

It is estimated that 0.1–0.3% of inhabitants in the Western world are affected by venous leg ulcers (VLUs; Nelzen, 2008). Ulcers that do not heal within six weeks are in several studies referred to as hard-to-heal (Fagerström et al., 2022; Forssgren et al., 2008; Forssgren & Nelzén, 2012). Previously, these ulcers were known as *chronic* (Dale et al., 1983). An ulcer’s healing time depends on its aetiology and can range from days to several decades (Oien & Forssell, 2013). This definition is not based on the cause of the ulcer (Vowden, 2011). Predisposed persons are at increased risk of getting ulcers and factors like diabetes, high blood pressure, and arterial and/or venous insufficiency increase the risk that an ulcer becomes hard to heal (Lindholm et al., 1992). Venous insufficiency causes blood to accumulate in the legs and feet, leading to oedema and local hypertension, which further exacerbates the circulatory insufficiency (Lindholm, 2018). About 50% of all leg and foot ulcers are caused by venous insufficiency, though the actual figure may be higher, since not all patients are diagnosed by physicians (Lindholm et al., 1992). A lacking or inaccurate diagnosis is likely to lead to longer healing time and increased suffering for the patient, since early management is crucial (Oien & Forssell, 2013). Varicose veins are also known to increase the risk that venous leg ulcers become hard to heal (National Health Service, 2019). Female sex, heredity, advanced age, pregnancy, and a job that involves standing are considered to be important causes of varicose veins (National Health Service, 2020). Knowledge of the importance of these mechanisms has been reported to be lacking among those affected by VLUs (Meulendijks et al., 2020).

A review has concluded that those affected by VLUs are typically aged over 65 years (Gethin et al., 2021). Advanced age plays a role in the development of VLUs (Meulendijks et al., 2019), and the average age at onset is nearly 80 years (Oien & Forssell, 2013). This also means that comorbidities are common (Gethin et al., 2021). Indeed, comorbidities such as obesity, coronary heart disease, angina, hypertension, dyspnoea, asthma, and diabetes have been reported (Padberg et al., 2004). Thus, some of those affected by VLUs are also affected by other hardships due to their medical conditions and/or advanced age, which can impact on daily life (Moffatt et al., 2004). The recurrence of VLUs is thought to be as high as 50% (Finlayson et al., 2018; McDaniel et al., 2002; Nelzén et al., 1997) and is likely to be frightening and undesirable among those affected by a VLU (Ebbeskog & Ekman, 2001).

For those living with active VLUs, pain is a part of daily life (Kapp et al., 2018; Upton & Andrews, 2013), as is interrupted sleep (Upton & Andrews, 2013), anxiety, and sometimes depression (Souza Nogueira et al., 2009). Social isolation is also common, and patients commonly report that VLUs are limiting in daily life (Briggs & Flemming, 2007; Kapp et al., 2018). Thus, the lifeworld with an ulcer is affected by hardships, but not much is known about the lifeworld after healing, since most research has focused on patients with *active* ulcers.

Though most cases of VLU can heal, the underlying problem in the veins predisposing individuals to develop VLUs is generally chronic. Studies have emphasized parameters facilitating life in a new context with a chronic illness, such as understanding and acceptance (Ambrosio et al., 2015; Chiaranai et al., 2018). It is also known that persons with chronic illnesses can experience a loss of stability in life. A changed balance and reduced control over life creates a new reality (Ambrosio et al., 2015). Since it is known that persons with chronic diseases may feel they have undergone a major change in life, it is of a great importance that we enhance knowledge about daily life for persons with healed VLUs. We intended to increase insights into their lifeworld, with a focus on lived experiences, and thereby get a chance to improve the situation among those affected. The lifeworld takes the individual’s whole situation—their perspective on their own situation and what is meaningful to them—into consideration (Dahlberg et al., 2008). The aim of this study was to explore and describe patients’ experiences of daily life after healing of a hard-to-heal venous leg ulcer.

## 2. Materials and methods

### 2.1. Study design

This study was conducted through interviews, with a basis in reflective lifeworld research (RLR), an approach with the goal to describe the essential structure of meanings and the constituents of a phenomenon—in this case: *daily life after healing of a hard-to-heal ulcer* (Dahlberg, 2014; Dahlberg et al., 2008). Reflective lifeworld research is based on the phenomenology of Husserl’s lifeworld theory and the theory of intentionality (Husserl, 1980) and the theory of the lived body by Merleau-Ponty (Merleau-Ponty, 1945/2018). This descriptive phenomenological design provides an opportunity to understand the lived experiences of persons with healed VLUs. Husserl’s theory of intentionality constitutes a solid foundation related to one’s own understanding. Concepts such as “bridling” and openness have an impact on a reflective lifeworld study, as the researcher “bridles” their pre-understanding and thereby remains open to what is actually said (Dahlberg et al., 2008). The importance of letting the phenomenon be boundless for as long as possible—or, as described by Dahlberg and Dahlberg: *not making definite what is indefinite* (Dahlberg & Dahlberg, 2003)*—*typifies the RLR approach.

### 2.2. Participants

Fifteen participants, six men and nine women, in southern Sweden were interviewed. Their age ranged from 58 to 86 years. The experiences of ulcers ranged from having had one ulcer, healed in months, to having had repeated ulcers over the course of half a century. The inclusion criteria were having been diagnosed with a venous ulcer (e.g., compression hosiery has been prescribed) with an ulcer duration of more than six weeks, having the cognitive ability to convey experiences, and having the ability to understand and speak Swedish. All participants lived in their own homes, with one of the men receiving home care. A variety among participants (e.g., as regards age, sex, social situation, and residence) was desired, broad lifeworld descriptions (Dahlberg et al., 2008).

### 2.3. Data collection

Suitable participants were first contacted and briefly informed about the study’s aim by the health care personnel who treated their ulcers. One pilot interview was conducted; this was included in the results section because of its richness. The interviews were carried out by the first author (MR) between June and November 2020. Due to travel restrictions and a focus on the security of the participants during the COVID-19 pandemic, the interviews were conducted via telephone. All participants chose to take the interview call in their personal home. All interviews started with an open-ended question: “Could you please describe your daily life?” In order to gain in-depth answers, follow-up questions such as “What does that mean for you?” or “How does that affect you?” were asked (Dahlberg et al., 2008). Interviews lasted between 17 and 81 minutes and were recorded and transcribed verbatim. Interview transcripts were not checked by participants. To supplement the recordings, field notes were taken. In the RLR approach, openness and curiosity towards the phenomenon under study requires interviews to be open, e.g., letting the phenomenon steer the conversation.

### 2.4. Data analysis

The interviews were transcribed verbatim and formed the basis for the analysis. The description of the phenomenon was obtained through the approach presented by Dahlberg et al. (Dahlberg et al., 2008). The focus in the RLR approach is the phenomenon under study. In order to let the phenomenon show itself, the researchers sought to stay open and bridled during the entire process, and nothing was taken for granted. Familiarization with the interviews, gained through multiple readings, gave an insight into the entirety of the material. Meaning units, related to the phenomenon under study, were retrieved from the transcribed interviews. Meanings were then clustered together, based on similarities and differences. Clusters were interrelated in order to find patterns of meanings within the phenomenon. A movement between the parts and the whole led to the emergence of the phenomenon’s essential meaning structure; a new wholeness was reached. The phenomenon’s essence could be described as its heart, at an abstract level, and is described and clarified by its constituents. Throughout the analysis, there was a focus on not making definite what was indefinite, which meant lingering before deciding upon a meaning (Dahlberg et al., 2008). Analysis was performed by the first and last author, and the process was discussed within the research group. The results are first presented through the essence of the phenomenon, *daily life after healing of a hard-to-heal ulcer*, and then through its constituents. Some quotes from the interviews are presented, to serve as examples.

### 2.6. Ethical considerations

This study complied with the principles outlined in the Declaration of Helsinki (World Medical Association, 2013). The study was approved by the Swedish Ethical Review Authority (dnr 2020–00965). As mentioned, patients were provided with written and verbal information about participating in the study before they gave written informed consent. All interviews were processed and stored securely, to protect the confidentiality of the participants. The researchers had no relationships to the participants and were not involved in their treatment.

## 3. Results/findings

### 3.1. The essence of the phenomenon: daily life after healing of a hard-to-heal ulcer

Daily life after healing means living with memories of the time with an ulcer. These memories are embedded in the lived body, and thoughts of the “then” affect the “now.” The ever-present memory bank includes memories of slow healing and physical and mental suffering.

The time with an ulcer was seen to influence the past and the present, as well as the future. The ulcer has left various types of scars on the lived body. Awareness of the own body instils calm, since bodily changes can be identified. A pragmatism rests over the wandering of life, where a road towards a new ulcer would be an unwanted detour. After healing, life has changed; the healed ulcer limited a life that had once been taken for granted. The protracted healing time also led to a desire to make life return to what it was before, or at least something similar. Being healed brought joy to daily life, despite other hardships.

A body beset by age and ailments can constitute an obstacle to carrying out self-care and activities of daily life. In a way, the ulcer was silent, since it had healed. The silence was the result of a symptom that was gone, though its causes might still remain. When an ulcer had healed, there could be a feeling of isolation due to having to take responsibility for one’s own future. The phenomenon encompassed a profound desire to manage the new situation through control. Sometimes, the body could offer a reassuring message on the measures taken. While the time with the ulcer was seen as a lost part of life, the future like a blank page: either there will be a new ulcer or not. This creates a balancing act for the future: on the one hand, there is concerns regarding the risk of new ulcers, and on the other hand, a belief in maintained health. Daily life has changed, and there is a new normal to relate to.

The essence could be further described through the following constituents: *Memories as an unbreakable link to the past, The ulcer is healed, yet always present, Wandering towards control over the lived body* and *Life in a new normal.*

### 3.2. Memories as an unbreakable link to the past

The close connection between the lived body and a person’s lifeworld meant that ulcers left a mark on life that remained even after healing. Daily life with an ulcer was described as a struggle in several ways; the period with the ulcer was characterized by hopelessness. The smell and sight of bodily fluids seeping out of the own body was an experience that could not be removed from the lifeworld. Experiencing oneself as disgusting remained like a scar deep inside.

*“There was so much fluid coming out of my foot, I don’t know how many towels we wrapped the leg with that night. And the next morning, I called them. (…) And it smelled really bad, like meat juice (…) and it seeped through everything.”* (Participant 9)

However, having contact with a specialist care unit was experienced as something positive. Positive experiences from health care were affirmed by several persons as being restorative: responsiveness, information, and confidence were cherished elements. Memories of a good caring relationship could brighten a dark period. However, some participants also referred to a relationship in which they were not listened to, which was a determining factor for who they would contact in the future. Among those who felt health care services had failed in their role, the experience of care was an additional suffering that still darkened their lifeworld.

Memories of frequent visits to the clinic, a struggle to manage to shower on the days that the wound was dressed, and spending nights awake because of pain had become part of the self. There was also the feeling that the own suffering had been unnecessary, since it had taken a long time to get the right help. This could bother some participants, even though they were now healed. These experiences could create the foundation for a fear of new ulcers. Although this fear was not a constant companion, it is always there as a shadow, ready to appear.

### 3.3. The ulcer is healed, yet always present

After several months with an ulcer, healing had occurred. This was seen as a joyous success. The healed ulcer was still present in daily life, with multiple consequences. A life after healing was a life with symptoms. Daily life was described as being filled with waiting to get well or at least experience some positive change. Positive changes in relation to physical impairments were a delight, but a sense of hollowness was never far away; changes in the wrong direction took a toll on the person and their daily life. In a way, the burden had changed guise; the ulcer was gone, but pain, itching, and eczema still tormented daily life. There was a constant awareness that the risk for new ulcers remained, even if there was hope, since treatment had been successful. Uncertainty regarding the reason behind the ulcer led to a feeling of powerlessness in daily life. Not knowing how to protect oneself against new ulcers increased this feeling of powerlessness.

*“I have to remind myself that I need the compression stockings. (…) I notice that my legs are swollen, and it hurts and so on. My skin doesn’t feel good, and I know it’s a strain on the skin.”* (Participant 3)

Other experiences, such as a difficult treatment period, could serve as reminders of the ulcer and connected it to the self, leading to a heightened sense of caution in relation to the own body. It was common to avoid situations that could increase the risk of a new ulcer. An ulcer was an event that was unwanted in the future, with the experience of the healed ulcer serving as a warning. There was an uncertainty regarding whether it would be possible to mentally deal with a new ulcer.

*“Actually, that’s what you are worried about and that I really don’t want to end up there again. (…) I’m avoiding it as much as I can. I don’t want it back.”* (Participant 13)

### 3.4. Wandering towards control over the lived body

Persons with healed ulcers undertake a wandering towards control in life. This wandering can be described as a constant search for an explanation of how all this could have happened. A reasonable and probable solution would be to identify the truth and make it a part of daily life. Meanwhile, daily life encompassed various methods to deal with decreased mobility due to pain, loss in muscle strength, or a failing sense of balance. These included using crutches, a wheelchair, or a walker, sometimes even if they were not prescribed for oneself; a strong desire for independent mobility created a need for aids. Elements that seemed to bestow some control over daily life were correct use of compression stockings, using moisturizing cream, or elevating one’s legs while seated. A sense of control could also be experienced in the form of loyalty towards health care personnel: “If I do what I can, he will do his best for me.” The wandering towards control could also cause an unpleasant feeling of stress, when control was lost.

*“I don’t think about the ulcer much, I just do what the doctor tells me, and in that sense am I loyal, so to speak.”* (Participant 12)

The wandering towards control could sometimes, at least in relation to outsiders, be labelled as a form of independence. The use of the less tight class II compression stockings, instead of the prescribed class III, offered a sense of control to the individual, who could now do as they liked. Independence was a recurring theme: an active choice not to use compression stockings in the summer or to use a cream intended only for acute treatment of thrombophlebitis over a long time period. There was a longing for proof that the measures were paying off: a moisturizer should provide visible, noticeable results and compression stockings should decrease swelling. The bodily results were seen as signs of control over the lived body.

*“For me, it’s so that I don’t get another leg ulcer, and that I’m in control when I apply moisturizer to my legs. Then I have control over it not coming back.”* (Participant 6)

### 3.5. Life in a new normal

There is an awareness of the fact that increasing age increased the risk of getting another ulcer. The ulcer and the healing time had taken their toll on the body, and as one woman described it:

*“As I see it, I’m really, really dilapidated.”* (Participant 10)

The ability to use the body as a tool to rediscover the world had changed. Some things that had previously been part of the daily routine or daily life could no longer be done. Physical activities and hobbies were no longer being carried out after healing. Even daily activities like gardening, shopping, or taking the stairs were associated with difficulty after healing; there was a boundary between then and now. Daily life had changed and many things had become impossible.

*“Every other Thursday, I go to the grocery store in town in the morning, we arrive at 8 am. I don’t know, life is not that eventful anymore.”* (Participant 14)

The lifeworld after healing encompassed decreased mobility and deformed toes, due to the compression stockings. Inabilities in daily life evoked emotions like frustration, irritation, and anger. It was hard to accept that everyday chores like dusting and window cleaning could no longer be done, at least not as before. After healing, some patients could not water their houseplants unless there were chairs waiting between all the windows, just in case. The lifeworld after healing was, to some extent, all about being prepared. A feeling of being compelled to change lifestyle was described as difficult to deal with.

Though anger and disappointment arose due to an inability to do what used to be normal, some activities were no longer carried out. In a way, daily life was governed by a reluctance to run the risk of getting another ulcer, with sacrifices being made to protect against such occurrence.

Life after healing could be experienced as a new lease on life, with a new reality to adapt to. As a remnant from the time with the ulcer, there is a feeling of being limited. Even though healing improved quality of life, it remained decreased due to a constant struggle between what the participants wanted to do and what they were physically able to do. To a large extent, daily life was characterized by loss and inability.

*“I guess it’s like learning to live with a handicap, eventually you adapt to the situation.”* (Participant 5)

Life involved of a degree of shame at the scarred body, compression stockings, and skin changes, which participants sometimes attempted to hide. The lifeworld after healing included changes in clothing and shoes. Combining shorts with compression stockings could be unthinkable, making long pants the only possibility, even on hot summer days.

After healing and the preceding time with the ulcer, life could be lonely. The experience of being incapacitated for a long time left traces on the lived body. A wife and husband might have to stop taking walks together, due to the new physical inability for one of them to keep up with the other. Not having driven a car for a long time might lead to a driving licence not having been renewed, so that life now involved always being a passenger.

Daily life could include a feeling of being dependent on others. Participants might have to ask their significant other for help around the house. Receiving municipal help at home might be necessary. Even if healing created liberty in the form of fewer visits to the clinic, the total disease burden might not have decreased. The aged body might be affected by other hardships. With or without an ulcer, life went on, with the loss of friends and family, progression of other diseases, and inevitable ageing. Life with a healed ulcer was just like normal—not the normal that it had been before, but a new normal.

## 4. Discussion

The aim of this phenomenological study was to describe the experiences of daily life after healing of a hard-to-heal venous leg ulcer. The ulcer had grown silent, since it had healed. While the ulcer was no longer there in a physical way, it remained vibrant in the lived body. Life had changed and a new normal emerged. Arman and Rhensfeldt (2012) have stated that life-changing events have the ability to alter one’s priorities in daily life. What has been important might lose or decrease in importance (Arman & Rhensfeldt, 2012). Persons with healed VLUs reported a newly discovered joy in being healed. Improvements in health may create new opportunities in life and thus a chance to change one’s life (Arman & Rhensfeldt, 2012). Though a change can be seen as an opportunity, it can also be experienced as a *loss*, rather than an active choice. Nevertheless, a new meaning might be created or at least initiated through the suffering of living with an ulcer (Arman & Rhensfeldt, 2006). For some reason, persons do not return to what life was like before the ulcer. A study with the aim to identify the relationship between the present and what the situation was like before an ulcer would therefore be of interest.

The participants’ lives contained memories from a, sometimes prolonged, period with a VLU. The inability to leave parts of one’s lifeworld behind can be described using the theory of Arman and Rhensfeldt (2006). In a way, suffering might be seen as a constant companion that never completely vanishes, even if it changes shape. The aim is always to create a balance in life—suffering must be bearable—and in order to achieve this, life must sometimes be reassessed and a new meaning found (Arman & Rhensfeldt, 2006). According to Arman and Rhensfeldt (2006), meaning is associated with hope and a future that offers something better. A new understanding of life and what it offers can create a meaning in life. A life without change is dark and characterized by unbearable suffering that is not associated with acceptance or openness (Arman & Rhensfeldt, 2006). But how does the loss of life as it was affect a person? Can the new, changed life induce new suffering, based on a lost capacity to continue with an activity or a hobby?

As mentioned, living with a healed ulcer means living a life different from the life before the ulcer. The reasons for that may vary. It seems that persons with chronic heart failure live with a constant fear of the future (Jeon et al., 2010). Falk et al. have stated that heart failure can cause persons to choose a “less complex and eventful life” (Falk et al., 2007), which also seems to be the case among persons with healed ulcers. Barello and Graffigna (2015) and Arman and Rhensfeldt (2006) have argued that a new meaning in life has to be created when a person is diagnosed with a chronic illness. Hobbies and activities that are discontinued during treatment are not always resumed after healing, sometimes being replaced by activities adapted for the new situation. Fatigue has been shown to be the main reason for this in persons with heart failure (Falk et al., 2007), but our study indicated that the main reason among persons with healed ulcer was a reluctance to risk getting a new ulcer. Still, the outcome is similar: a restricted life. Limitations in life and activities are also seen in persons receiving haemodialysis (Chiaranai, 2016). We argue that persons with a healed ulcer share some experiences with persons with other chronic illnesses. This is supported by a study encompassing multiple chronic illnesses, in which the researchers stated that those affected often described to their lives as restricted (Chiaranai et al., 2018).

Life after healing also consisted of a wandering towards control in life. Brännström et al. stated that the unpredictability of living with a chronic illness affected life, due to never knowing if or how the condition might worsen. In the research of Brännström et al., it seemed that patients had some degree of hope regarding the future, such as longing for summertime when it would be possible to get out (Brännström et al., 2006). Even if the future was rather uncertain for the persons with VLU, it seemed that the new life after healing was associated not only with anxiety, but also with a belief that they might stay healthy. However, the present study did not explore the future and not much was said about it, beyond the hope not to get another ulcer. This may mirror what was found by Barello and Graffigna, who stated that patients might lack confidence in the future, causing an incapacity to make plans for it (Barello & Graffigna, 2015). Bonino stated that chronically ill persons need to adjust not only their goals, but also their entire identity, in the same way they would in ageing (Bonino, 2021). As mentioned before, persons affected by VLUs are often older adults, which would mean such changes are necessary for several reasons.

In the present study, persons seemed to see different dimensions in their lives—the ulcer might either return or not—and these thoughts did not seem to paralyze them in daily life. Rhensfeldt and Eriksson have argued that there is a need for two poles in life (hope-hopelessness, anxiety-satisfaction), creating an opportunity to move forward (Arman & Rhensfeldt, 2006). The participants had confidence in the future, a confidence that could be supported by their managing to return to daily life as it was before the ulcer. Health care personnel could have a major role to play in this during the long treatment.

Participants in the present study referred to their lives as *normal*, but scratching the surface revealed something else. This made us ask ourselves: *what is a normal life?* Robinson has stated that *the normal* in persons’ lives tends to change over time; illness imposes a *new normal*, that comes to be accepted with time. Robinson also mentioned that by concentrating on living normally, setbacks and issues could be disregarded and would lose their importance in daily life. For example, frequent hospitalizations or chronic pain could be accepted, since there they were an inevitable part of life (Robinson, 1993) and had become the new normal. This could give a sense of control in a new life context, which might be affected by the loss of cherished activities. Barello and Graffigna also emphasized that there is an underlying desire to be normal, even when life may take a new turn (Barello & Graffigna, 2015). The ties between the self’s history, present, and future were apparent in this study. Merleau-Ponty stated that the human body is a tool for accessing the world and that it consists of experiences gathered over time. The body as an *experiencer* entails that we can never free ourselves from our past—the body is ever-present in what happens in life (Merleau-Ponty, 1945/2018). As a basis for reflective lifeworld research, Dahlberg et al. stated that the lifeworld consists of all the experiences in a person’s life, with all the lived experiences influencing the perception of one’s own situation (Dahlberg et al., 2008). With this in mind, it is understandable that one can never free oneself from one’s history. The experience of living *with* an ulcer leaves a mark on a person even after healing: memories are forever. But as Bonino stated: life should not be survived, but lived (Bonino, 2021). Active ulcers are known to have physical, psychological, and social impacts on daily life (Isaac & Watson, 2016) and this study serves as a reminder that healing does not automatically negate these effects.

Lived experiences of persons with healed ulcers might form a basis for information to persons with active ulcers. Taking the whole lifeworld into consideration must be an objective for the health care personnel caring for these persons, to prepare them for a future and daily life without an ulcer. We suggest more research among persons who have had a VLU that has healed, to determine whether counselling or physiological support should be included in ulcer management.

### 4.1. Strengths and weaknesses

There are some tools in the RLR that have been applied throughout the research process. Openness, flexibility, and the bridling approach (Dahlberg et al., 2008) helped us in striving for objectivity and curiosity towards the phenomenon. The research group openly discussed the results during the process, creating a flexibility that allowed us to constantly move back and forth between the transcripts and the essence. The results are valid, thanks to the sensitivity and openness during the entire process, not least in the development of the essence and its constituents. Though it is said that phenomenological results are never final, but rather eternally evolving with the questions asked and the context, a consistent desire to stay focused on the phenomenon at hand, instead of the subject, results in an essence that illuminates the entire phenomenon being studied (Dahlberg et al., 2008). The essential description of a phenomenon is generalizable—otherwise it would not be essential—and thus transferable to other areas (Van Wijngaarden et al., 2017).

Reflective lifeworld research interviews should be held in person, but since the prevailing situation did not permit that, the decision was made to use the telephone as a means to gather lived experiences in this study. There are arguments that rich data—provided by participants, with a focus on the phenomenon—are more important than the means of the handover itself (Dahlberg et al., 2008). Telephone interviews may offer a greater perceived anonymity, making it easier to speak openly (Trier-Bieniek, 2012). There is also a belief that there are no differences between face-to-face and telephone interviews as regards the nature or depth of material (Sturges & Hanrahan, 2004). However, a sensitivity to pauses and changes in voice or mood can help the interviewer stay focused on the participant’s feelings during an interview. When scheduling the interviews, care was taken to ensure enough time with each participant.

Conducting research during a pandemic had some consequences. Telephone interviews were an obvious choice, as there was no possibility for the researchers to visit health care facilities. Several participants expressed pleasure at getting the opportunity to share their experiences and highlighted the importance of this.

This sample of fifteen participants, who varied as regards sex, age, ulcer duration, social life, and residence, provided detailed narratives. A weakness that has to be recognized is the fact that none on the participants had a non-Swedish background. However, the authors have made the assessment that this study, based on the methodological ideas of the RLR approach, has provided the field with important findings—not only for researchers, but also for health care personnel, students within nursing, and persons affected by ulcers.

Some of the researchers’ past experiences must be mentioned: GL has scientific experience within the field of diabetic ulcers. HT and CF are involved in an ongoing research project in VLUs. MR has no experience within the field of ulcers.

## 5. Conclusion

The phenomenon of daily life after healing of a venous hard-to-heal leg ulcer means living in a new normal, characterized as somewhat restricted. Though some referred to their daily life as normal, life had not yet returned to what it was before. Some activities or hobbies, that had been a part of the self before the ulcer, had been terminated. Daily life after a healed ulcer was thus comparable to daily life for persons affected by other chronic illnesses. The lifeworld with a healed ulcer meant living with an obvious impact from the ulcer. The period with an ulcer, which was sometimes prolonged and always entailed hardship, had become part of life and could not be expunged from the present. Thus, ulcers leave more than just a physically scarred body. This study complemented our knowledge by revealing that daily life was shaken to its core, meaning that living with a hard-to-heal ulcer and all that this entailed had left a lasting legacy.

## 6. Implications

This study points out a need for health care personnel to prepare patients for a life without an ulcer and its results have the potential to enhance knowledge about lived experiences after healing an ulcer and to serve as decision support. There is a need to further examine the mechanisms behind a change in a person’s daily life, i.e., what or who creates that change. The findings of this study contribute with lived experiences regarding the clinical nursing of VLUs. These experiences should be considered when supporting, treating, and following up patients with VLUs. Patients could benefit from being given information regarding the ulcer during treatment, to make informed choices concerning their own health.

## References

[cit0001] Ambrosio, L., García, J., Riverol, M., Anaut, S., Ayesa, S., Sesma, M., Caparrós, N., & Portillo, M. (2015). Living with chronic illness in adults: A concept analysis. *Journal of Clinical Nursing* 24 17–18 , 24-2357-67 doi:10.1111/jocn.12827. 25951949

[cit0002] Arman, M., & Rhensfeldt, A. (2006). *Caring that relieves suffering. Ethics in care. [Vårdande som lindrar lidande. Etik i vårdandet]* (Stockholm: Liber).

[cit0003] Arman, M., & Rhensfeldt, A. (2012). The existential dressing - existential caring after a disaster. *[DEF - det existentiella förbandet: Existentiellt omhändertagande efter katastrof]*. Stockholm: Liber.

[cit0004] Barello, S., & Graffigna, G. (2015). Engaging patients to recover life projectuality: An Italian cross-disease framework. *Quality of Life Research*, 24(5), 1087–9. 10.1007/s11136-014-0846-x 25373927

[cit0005] Bonino, S. (2021). *Coping with chronic illess. Theories, issues and lived experiences*. London: Routledge.

[cit0006] Brännström, M., Ekman, I., Norberg, A., Boman, K., & Strandberg, G. (2006). Living with severe chronic heart failure in palliative advanced home care. *European Journal of Cardiovascular Nursing*, 5(4), 295–302. 10.1016/j.ejcnurse.2006.01.006 16546447

[cit0007] Briggs, M., & Flemming, K. (2007). Living with leg ulceration: A synthesis of qualitative research. *Journal of Advanced Nursing*, 59(4), 319–328. 10.1111/j.1365-2648.2007.04348.x 17608682

[cit0008] Chiaranai, C. (2016). The lived experience of patients receiving hemodialysis treatment for end-stage renal risease: A qualitative study. *Journal of Nursing Research*, 24(2), 101–108. 10.1097/jnr.0000000000000100 26275156

[cit0009] Chiaranai, C., Chularee, S., & Srithongluang, S. (2018). Older people living with chronic illness. *Geriatric Nursing*, 39(5), 513–520. 10.1016/j.gerinurse.2018.02.004 29598960

[cit0010] Dahlberg, K. (2014). *Examine health and caring [Att undersöka hälsa & vårdande]*. Stockholm: Natur & Kultur.

[cit0011] Dahlberg, H., & Dahlberg, K. (2003). To not make definite what is indefinite: A phenomenological analysis of perception and its epistemological consequences in human science research. *The Humanistic Psychologist*, 31(4), 34–50. 10.1080/08873267.2003.9986933

[cit0012] Dahlberg, K., Dahlberg, H., & Nyström, M. (2008). *Reflective lifeworld research*. Lund: Studentlitteratur.

[cit0013] Dale, J. J., Callam, M. J., Ruckley, C. V., Harper, D. R., & Berrey, P. N. (1983). Chronic ulcers of the leg: A study of prevalence in a Scottish community. *Health Bulletin (Edinburgh)*, 41(6), 310–314. 6654677

[cit0014] Ebbeskog, B., & Ekman, S.-L. (2001). Elderly persons’ experiences of living with venous leg ulcer: Living in a dialectal relationship between freedom and imprisonment. *Scandinavian Journal of Caring Sciences*, 15(3), 235–243. 10.1046/j.1471-6712.2001.00018.x 11564231

[cit0015] Fagerström, C., Wickström, H., & Tuvesson, H. (2022). Still engaged – Healthcare staff’s engagement when introducing a new eHealth solution for wound management: A qualitative study. *BMC Health Services Research*, 22(1), 103. 10.1186/s12913-022-07515-3 35078483 PMC8788143

[cit0016] Falk, K., Granger, B. B., Swedberg, K., & Ekman, I. (2007). Breaking the vicious circle of fatigue in patients with chronic heart failure. *Qualitative Health Research*, 17(8), 1020–1027. 10.1177/1049732307306914 17928476

[cit0017] Finlayson, K. J., Parker, C. N., Miller, C., Gibb, M., Kapp, S., Ogrin, R., Anderson, J., Coleman, K., Smith, D., & Edwards, H. E. (2018). Predicting the likelihood of venous leg ulcer recurrence: The diagnostic accuracy of a newly developed risk assessment tool. *International Wound Journal*, 15(5), 686–694. 10.1111/iwj.12911 29536629 PMC7949606

[cit0018] Forssgren, A., Fransson, I., & Nelzén, O. (2008). Leg ulcer point prevalence can be decreased by broad-scale intervention: A follow-up cross-sectional study of a defined geographical population. *Acta Dermato-Venereologica*, 88(3), 252–256. 10.2340/00015555-0433 18480924

[cit0019] Forssgren, A., & Nelzén, O. (2012). Changes in the aetiological spectrum of leg ulcers after a broad-scale intervention in a defined geographical population in Sweden. *European Journal of Vascular and Endovascular Surgery*, 44(5), 498–503. 10.1016/j.ejvs.2012.07.016 22925998

[cit0020] Gethin, G., Vellinga, A., Tawfick, W., O’Loughlin, A., McIntosh, C., Mac Gilchrist, C., Murphy, L., Ejiugwo, M., O’Regan, M., Cameron, A., & Ivory, J. D. (2021). The profile of patients with venous leg ulcers: A systematic review and global perspective. *Journal of Tissue Viability*, 30(1), 78–88. 10.1016/j.jtv.2020.08.003 32839066

[cit0021] Husserl, E. (1980). *Ideas pertaining to a pure phenomenology and to a phenomenological philosophy*. Martinus Nijhoff publishers.

[cit0022] Isaac, A., & Watson, C. (2016). How venous leg ulcers affect quality of life. *Primary Health Care*, 26(3), 18–23. 10.7748/phc.26.3.18.s30

[cit0023] Jeon, Y.-H., Kraus, S., Jowsey, T., & Glasgow, N. (2010). The experience of living with chronic heart failure: A narrative review of qualitative studies. *BMC Health Services Research*, 10(1), 77. 10.1186/1472-6963-10-77 20331904 PMC2851714

[cit0024] Kapp, S., Miller, C., & Santamaria, N. (2018). The quality of life of people who have chronic wounds and who self-treat. *Journal of Clinical Nursing*, 27(1–2), 182–192. 10.1111/jocn.13870 28493644

[cit0025] Lindholm, C. (2018). *Ulcers [Sår]*. Lund: Studentlitteratur.

[cit0026] Lindholm, C., Bjellerup, M., Christensen, O. B., & Zederfeldt, B. (1992). A demographic survey of leg and foot ulcer patients in a defined population. *Acta Dermto-Venereologica*, 72(3), 227–230. 10.2340/0001555572227230. 1357869

[cit0027] McDaniel, H. B., Marston, W. A., Farber, M. A., Mendes, R. R., Owens, L. V., Young, M. L., Daniel, P. F., & Keagy, B. A. (2002). Recurrence of chronic venous ulcers on the basis of clinical, etiologic, anatomic, and pathophysiologic criteria and air plethysmography. *Journal of Vascular Surgery*, 35(4), 723–728. 10.1067/mva.2002.121128 11932670

[cit0028] Merleau-Ponty, M. (1945/2018). *Phenomenology of perception*. Routledge & Kegan Paul.

[cit0029] Meulendijks, A. M., de Vries, F. M. C., van Dooren, A. A., Schuurmans, M. J., & Neumann, H. A. M. (2019). A systematic review on risk factors in developing a first-time Venous Leg Ulcer. *The Journal of the European Academy of Dermatology and Venereology*, 33(7), 1241–1248. 10.1111/jdv.15343 30422345

[cit0030] Meulendijks, A. M., Welbie, M., Tjin, E. P. M., Schoonhoven, L., & Neumann, H. A. M. (2020). A qualitative study on the patient’s narrative in the progression of chronic venous disease into a first venous leg ulcer: A series of events. *British Journal of Dermatology*, 183(2), 332–339. 10.1111/bjd.18640 31677155

[cit0031] Moffatt, C., Franks, P., Doherty, D. C., Martin, R., Blewett, R., & Ross, F. (2004). Prevalence of leg ulcerations in a London population. *QJM: Monthly Journal of the Association of Physicians*, 97(7), 431–437. 10.1093/qjmed/hch075 15208431

[cit0032] National Health Service. (2019). *Causes - Venous leg ulcer*. Retrieved 2020-10-09 from https://www.nhs.uk/conditions/leg-ulcer/causes/

[cit0033] National Health Service. (2020). *Causes - Varicose veins*. Retrieved 2020-10-09 from https://www.nhs.uk/conditions/varicose-veins/causes/

[cit0034] Nelzen, O. (2008). Prevalence of venous leg ulcer: The importance of the data collection method. *Phlebolymphology*, 15(4), 143–150. http://www.phlebolymphology.org/2009/04/prevalence-of-venous-leg-ulcer-the-importance-of-the-data-collection-method/

[cit0035] Nelzén, O., Bergqvist, D., & Lindhagen, A. (1997). Long-term prognosis for patients with chronic leg ulcers: A prospective cohort study. *European Journal of Vascular and Endovascular Surgery*, 13(5), 500–508. 10.1016/S1078-5884(97)80179-7 9166274

[cit0036] Oien, R. F., & Forssell, H. W. (2013). Ulcer healing time and antibiotic treatment before and after the introduction of the registry of ulcer treatment: An improvement project in a national quality registry in Sweden. *BMJ Open*, 3(8), e003091–e003091. 10.1136/bmjopen-2013-003091 PMC3753517 23959752

[cit0037] Padberg, F. T., Johnston, M. V., & Sisto, S. A. (2004). Structured exercise improves calf muscle pump function in chronic venous insufficiency: A randomized trial. *Journal of Vascular Surgery*, 39(1), 79–87. 10.1016/j.jvs.2003.09.036 14718821

[cit0038] Robinson, C. (1993). Managing Life with a Chronic Condition: The Story of Normalization. *Qualitative Health Research*, 3(1), 6–28. 10.1177/104973239300300102 8457793

[cit0039] Souza Nogueira, G., Rodrigues Zanin, C., Miyazaki, M. C., & Pereira de Godoy, J. M. (2009). Venous leg ulcers and emotional consequences. *The International Journal of Lower Extremity Wounds*, 8(4), 194–196. 10.1177/1534734609350548 19934181

[cit0040] Sturges, J. E., & Hanrahan, K. J. (2004). Comparing telephone and face-to-face qualitative interviewing: A research note. *Qualitative Research*, 4(1), 107–118. 10.1177/1468794104041110

[cit0041] Trier-Bieniek, A. (2012). Framing the telephone interview as a participant-centred tool for qualitative research: A methodological discussion. *Qualitative Research*, 12(6), 630–644. 10.1177/1468794112439005

[cit0042] Upton, D., & Andrews, A. (2013). Sleep disruption in patients with chronic leg ulcers. *Journal of Wound Care*, 22(8), 389–394. 10.12968/jowc.2013.22.8.389 23924837

[cit0043] van Wijngaarden, E., Meide, H. V., & Dahlberg, K. (2017). Researching health care as a meaningful practice: toward a nondualistic view on evidence for qualitative research. *Qualitative Health Research*, 27(11), 1738–1747. 10.1177/1049732317711133 28799478

[cit0044] Vowden, P. (2011). Hard-to-heal wounds made Easy. *Wounds International* 2 1 . https://www.woundsinternational.com/uploads/resources/8b209a588e74f81cc9dda6ed140a3c85.pdf

[cit0045] World Medical Association. (2013). World medical association declaration of helsinki: ethical principles for medical research involving human subjects Journal of the American Medical Association 310 20 2191–2194. 10.1001/jama.2013.281053. 24141714

